# Proposal for optimal placement platform of bikes using queueing networks

**DOI:** 10.1186/s40064-016-3703-2

**Published:** 2016-12-03

**Authors:** Shinya Mizuno, Shogo Iwamoto, Mutsumi Seki, Naokazu Yamaki

**Affiliations:** 1 Shizuoka Institute of Science and Technology, 2200-2 Toyosawa, Fukuroi, Shizuoka 437-8555 Japan; 2 Regius Ltd., 3-34-50 Handayama, HIgashi-ku, Hamamatsu, Shizuoka 431-3125 Japan; 3 Shizuoka University, 836 Ohya, Suruga-ku, Shizuoka, 422-8529 Japan

**Keywords:** Closed queueing network, Optimization, Gravity model, Cloud computing

## Abstract

In recent social experiments, rental motorbikes and rental bicycles have been arranged at nodes, and environments where users can ride these bikes have been improved. When people borrow bikes, they return them to nearby nodes. Some experiments have been conducted using the models of Hamachari of Yokohama, the Niigata Rental Cycle, and Bicing. However, from these experiments, the effectiveness of distributing bikes was unclear, and many models were discontinued midway. Thus, we need to consider whether these models are effectively designed to represent the distribution system. Therefore, we construct a model to arrange the nodes for distributing bikes using a queueing network. To adopt realistic values for our model, we use the Google Maps application program interface. Thus, we can easily obtain values of distance and transit time between nodes in various places in the world. Moreover, we apply the distribution of a population to a gravity model and we compute the effective transition probability for this queueing network. If the arrangement of the nodes and number of bikes at each node is known, we can precisely design the system. We illustrate our system using convenience stores as nodes and optimize the node configuration. As a result, we can optimize simultaneously the number of nodes, node places, and number of bikes for each node, and we can construct a base for a rental cycle business to use our system.

## Background

Recently, social experiments have been conducted using rental bikes available at distribution nodes. When people borrow bikes, they return them to nearby nodes. Table 1 shows example models, such as Hamatyari of Yokohama (Kikuchi 2011), the Niigata Rental Cycle, and Bicing of Barcelona (Takami et al. 2011). However, many problems have been reported from these experiments. Moreover, it is costly to run these operations. The results of these experiments are obscure regarding the system construction, including the number of bikes, nodes that should be used, and placement of nodes. Because the selection of the parameters of a system depends on the person designing it, the resulting system may not be optimal.

**Table 1:** Examples of rental bicycle systems

City	Name of rental bicycle	Number of nodes	Number of bikes
Niigata, Japan	Niigata Rental Cycle (4/2003–present)	20	164
Edogawa-ku, Japan	E-Cycle social experiment (9/2009–present)	3	400
Yokohama, Japan	Yokohama city community cycle (10/2009–11/2009)	10	100
Toyama, Japan	Aville (3/2011–6/2011)	15	150
Sakai, Japan	Sakai community cycle (9/2011–present)	4	450
Barcelona, Spain	Bicing (12/2007–present)	250	3000
Paris, France	Velib (7/2007–present)	1500	20,000
London, UK	London cycle Hire scheme (7/2010–present)	400	6000
Lyon, France	Vélo’v (5/2005–present)	250	3400
Madison, WI, USA	Madison B-cycle (3/2013–present)	33	300

In this paper, we attempt to solve problems such as where to place nodes, how many nodes should be prepared, and how many bikes should be available for each node. We formulate a model using the queueing network (Miyazawa 1993, 2006), and this model is calculated using mathematical analysis.

Previous fundamental research on queueing networks includes that of Gordon and Newell (1967), who proposed improvements to Jackson-style closed queueing networks (Jackson 1957). Baskett et al. (1975) developed BCMP networks, which are a general queueing model with complex classes and arbitrary service distribution. We can build a flexible model using these approaches. It is also important to compute a characteristic value for a closed queueing network; we often use the convolution algorithm (Buzen 1973) and mean value analysis (Reiser and Lavenberg 1980).

Applied works include George and Xia (2011) and Waserhole and Jost (2016), who describe vehicle rental systems using closed queuing networks. In particular, Waserhole and Jost discuss optimization over nonstationary demands. Their model needs some additional work, because it does not include the ability to set vehicles at a station. Therefore, we construct a model with vehicle capacity. In another approach to bike-sharing systems, Etienne and Latifa (2014) looked at mobility patterns using model-based clustering methodology and analyzed 2,500,000 trip data points. Boyac et al. (2015) proposed an optimization framework for car sharing in Nice, France. It would be interesting to analyze their data using the approach of Etienne and Latifa.

Similar bike rental systems are now being used all over the world. In Japan, the Ministry of Economy, Trade and Industry has authorized a plan to utilize electrically assisted bicycle-drawn carts for delivery businesses (Ministry of Economy, Trade and Industry 2014). There are various such plans as social experiments in Japan (Yamakawa 1992; Abe and Kawashima 2003; Miida 2002; Kawamoto 2007). Zhang et al. (2016) analyzed China’s model (in Ningbo, Hangzhou, and Beijing) and described the rental station planning of bicycle sharing systems, as well as the allocation, operation, and dispatch of public bicycles (Zhang et al. 2016). Aeschbach et al. (2015) examined London’s Barclays Cycle Hire. Here, we consider improving these models with a generic rental bike system that does not depend on specific areas.

Using our model, we can easily visualize the settings of the system as they change with time. Our method for designing bike distribution systems does not depend on the country or the area being deployed. Thus, as an example, we use convenience stores as nodes to distribute bikes. Moreover, we use the Google Maps application program interface (API) to obtain parameters, such as transit time and distance between nodes.

## Modeling using closed queueing networks

In this section, we consider how to arrange bikes at nodes. We solve this problem using closed queuing networks. First, we assume that the number of bikes is equal to the number of users in the system. Then, we formulate a model as follows:

1. Our distribution system contains one class of bikes.
2. The system contains *K* nodes.
3. *N* is the total number of bikes in this system. *N* is limited. The number of bikes at node *k* is denoted by $n_k$, where $N = \sum _{k=1}^K n_k$.
4. The service period at node *k* follows an exponential distribution and has a mean of $\frac{1}{\mu _k}$.
5. $\alpha _k$ is the arrival rate of bikes that have reached node *k* from other nodes in the system.
6. $p_{i,j}$ is the probability that a bike served at node *i* travels to node *j*, such that $1\,\le\,i,\,j\,\le\,K$, $p_{i,j}\,\ge\,0$, $\sum _{j=1}^K p_{i,j} = 1$.

**Fig. 1:**
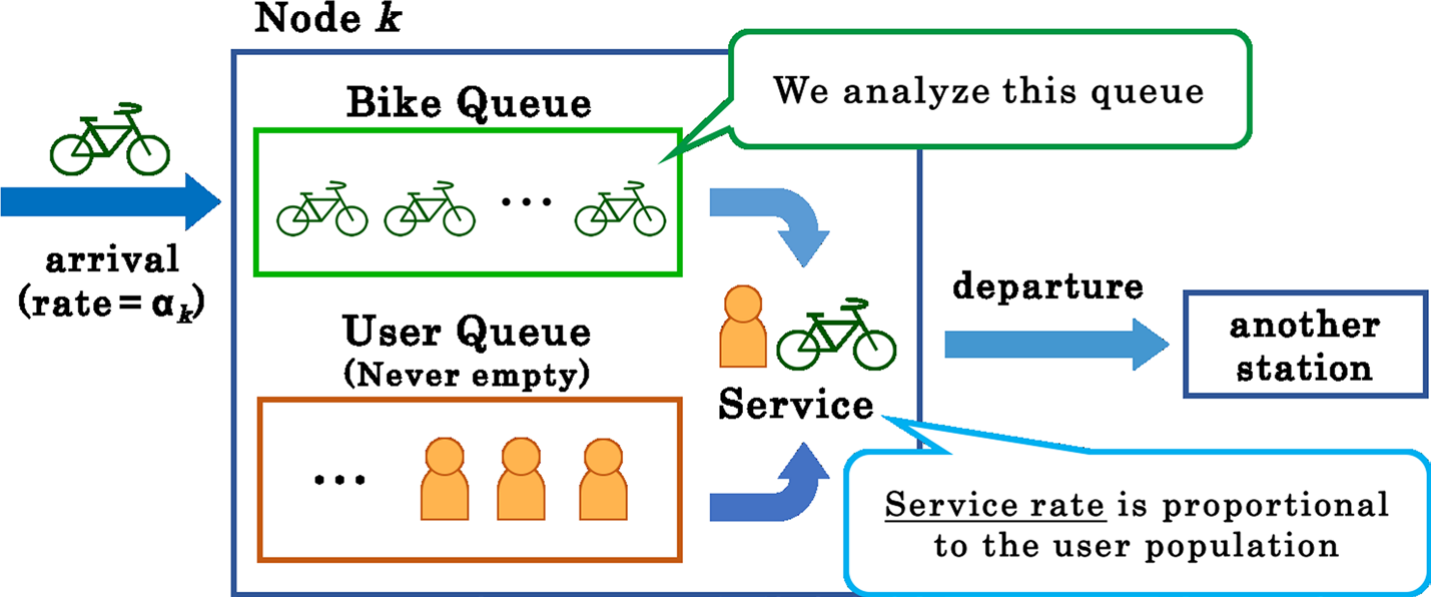
Treated object in this closed queueing network model

Regarding step 4, as illustrated in Fig. 1, we assume the number of bikes is equal to the number of customers, as in usual queueing models. We also assume that the number of users that they want to use bikes is unlimited, so we adjust the duration of bike visits using the service rate of each node. We take the service rate to be proportional to the rate of use for users at node *k*. If there are few users at node *k*, bikes are not used by many users, so they stay for a long time at the node. In other words, the service rate is low. In this model, we assume an *M*/*M*/1 closed queueing network. Thus, we have a server at each node. We cannot lend two or more bikes simultaneously. However, we can extend this model to a Gordon–Newell network (Gordon and Newell 1967) easily, and we can lend a number of bikes simultaneously that is the same as the number of servers prepared at each node.

$\alpha _k$ is the solution of the traffic equation,

1 $$\begin{aligned} x_k = \sum _{j = 1}^{K} x_j p_{j,k},\quad k = 1,2,\ldots , K. \end{aligned}$$

and we insert when we compute the closed queueing network. Moreover, we set $\sigma _k = \frac{\alpha _k}{\mu _k},\,k=1,2,\ldots ,K$. The probability that $n_k$ bikes exist at node *k* is obtained according to the stationary distribution

2 $$\begin{aligned} \pi (n_1, n_2, \ldots , n_k)= & {}\, \frac{1}{G(N,K)}\prod _{k=1}^K \sigma _k^{n_k}, \nonumber \\ where \quad G(N,K)= & {} \sum _{n_1+\cdots +n_K = N}\prod _{k=1}^K \sigma _k^{n_k}. \end{aligned}$$

Moreover, the transition probability $p_{i,j}$ is computed according to the following gravity model (Anderson 2011; Flowerdew and Aitkin 1982; Carrère 2006; Yeates 1969):

- $f_{i,j}$: Movement from area *i* to *j*, which we obtain from (3),
- $q_{i,j}$: Total movement from area *i* to area *j*,
- $r_{j,i}$: Total movement from area *j* to area *i*,
- $s_{i,j}$: Absolute value of the elevation difference between nodes *i* and *j*,
- $d_{i,j}$: Distance between areas *i* and *j*,
- *C*: Constant value of the gravity normalization model,

and

3 $$\begin{aligned} f_{i,j} = C \frac{q_{i,j}^{\alpha } r_{j,i}^{\beta } s_{i,j}^{\gamma }}{d_{i,j}^{\eta }}, \quad i,j = 1,2,\ldots , K. \end{aligned}$$

Note that $q_i$ and $r_j$ are both asymmetric: riders tend to go to more popular nodes from less popular one. So, $q_i$ and $r_j$ need information about direction of movement. We also often need to consider the elevation of each node. If this is of no concern in an area, we set $\gamma$ to 0. If we consider $d_{i,j}$ to be important, then we increase the distance parameter $\eta$. These parameters indicate what we emphasize, either distance or population, for the transition probability in this model.

Normalizing $f_{i,j}$, we obtain the transition probability $p_{i,j}$. If

- $L_k$: Number of bikes in the system at node *k*,
- $CP_k$: Capacity of node *k*,

then the problem of assigning bikes to nodes can be formulated as follows:

4 $$\begin{aligned}&minimize \nonumber \\&\sum _{k=1}^K (\left| L_k - CP_k \right| + PT_k \cdot I(\overline{A}_k)), \end{aligned}$$

where event $A_k$ indicates that it satisfied the condition for the number of bikes at node *k*. In other words, $A_k$ is an appropriate number of bikes for the node, for example, less than capacity while maintaining the number of bikes below the minimum requirement at *k*. $PT_k$ is the penalty cost. *I*(*E*) is an indicator function; if an event *E* is satisfied, this function returns 1 and if not, it returns 0. We define the objective function (4). In this equation, we use $L_k$, which denotes the number of bikes in the system at node *k*. Thus, we optimize the bike arrangement so as not to crowd the bikes at a node. If the number of bikes at node *k* is not within the safety range described by event $A_k$, we must add the penalty $PT_k$ to the objective function.

We know that the problem of bikes tending to converge at a node occurs. We must transport bikes to distribute them at each node. The objective function (4) indicates that bikes are distributed as efficiently as possible.

## Configuration of the proposed system

**Fig. 2:**
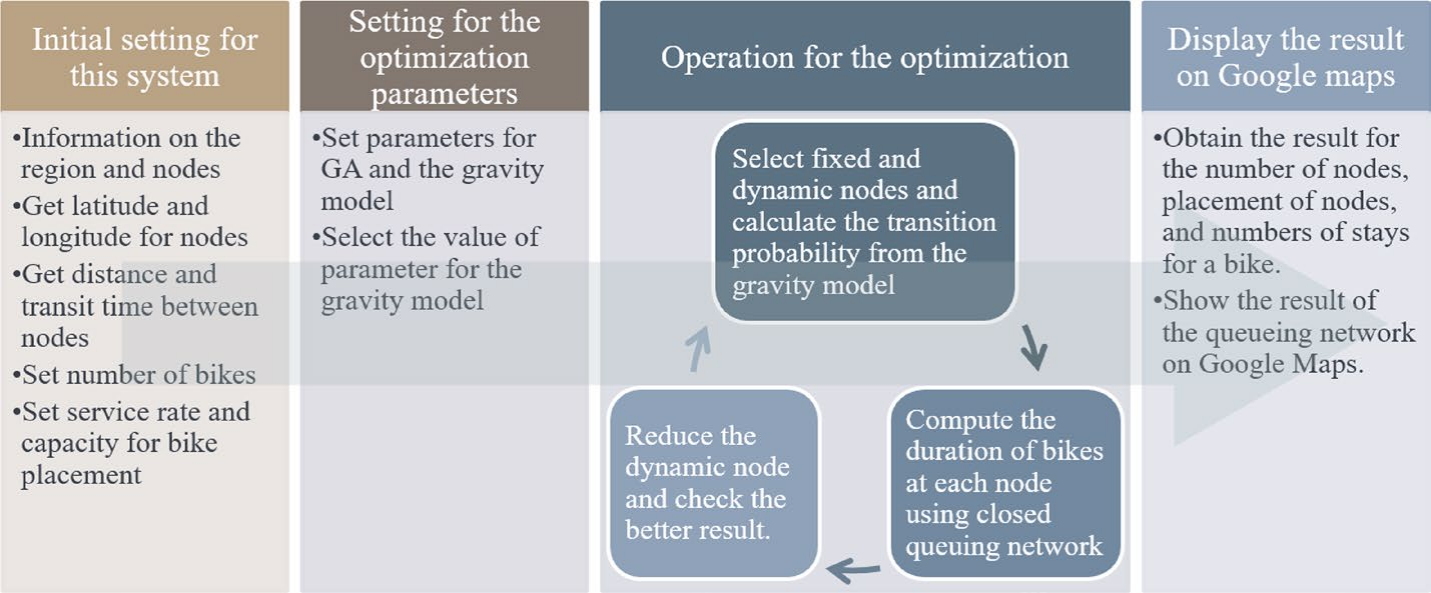
Flow of the proposed system

In Fig. 2, we show the procedure used to compute the optimal placement of the nodes in our system. A region is defined as the entire target area and an area has a postal code and population. We obtain the transition probability for the gravity model from (3), using the area as the minimum unit of the gravity model.

### Initial settings

Before proceeding with the computations, we select the initial settings. The database of our system uses two tables for initial settings. In the first table, we enter the postal code data, which consists of the postal code, state name, region name, city name, town name, and population, as shown in Table 2.

**Table 2:** Example of postal code data used by our system

Postal code	State name	Region name	City name	Town name	Population
4300805	Shizuoka	Koutou District	Naka-ku Hamamatsu-shi	Aioi-cho	858
4338111	Shizuoka	Hagioka District	Naka-ku Hamamatsu-shi	Aoinishi	9814
4338114	Shizuoka	Hagioka District	Naka-ku Hamamatsu-shi	Aoihigashi	2159
4328043	Shizuoka	Kousai District	Naka-ku Hamamatsu-shi	Asada-cho	813

We input information data for the distribution nodes, which consists of the node name, postal code, address, latitude, longitude, service rate, and capacity, as shown in Table 3.

**Table 3:** Example of information data for the nodes used by our system

ID	Node name	Postal code, address	Latitude, longitude	Service rate	Capacity
1	Hamamatsu training school of information	4300929, Naka-ku Hamamatsu-shi, Shizuoka	34.7071129, 137.7409474	5.0	10
2	Thanks Hamamatsu Act Street	4300928, Naka-ku Hamamatsu-shi, Shizuoka	34.7084987, 137.7340032	5.0	10
3	Thanks Hamamatsu Sumiyoshi	4300906, Naka-ku Hamamatsu-shi, Shizuoka	34.7309325, 137.7241608	5.0	10
4	Thanks Hamamatsu Wagou	4338125, Naka-ku Hamamatsu-shi, Shizuoka	34.7392201, 137.7079812	5.0	10

**Fig. 3:**
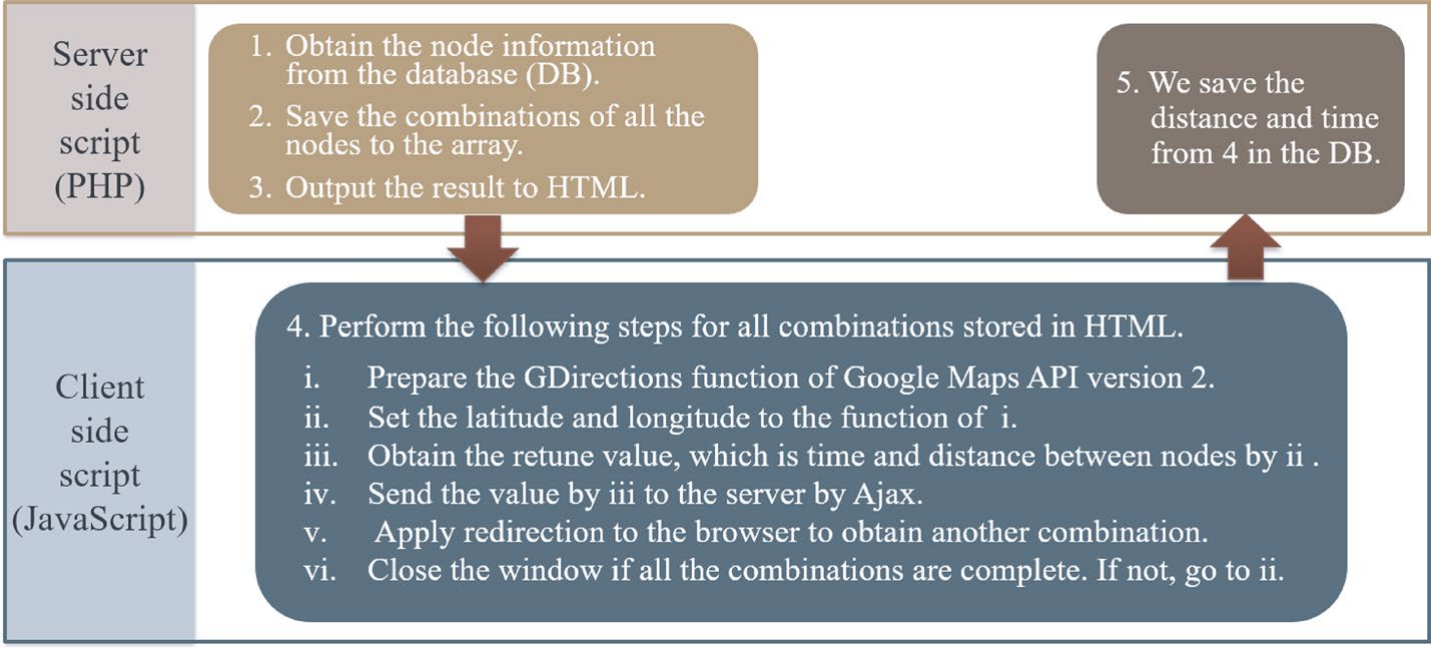
Parameter acquisition process for all nodes

Given the node information, we can compute the distance and transit time between nodes *i* and *j*. In Fig. 3, we show the procedure to acquire information for all nodes. Commercially, we use the Google Maps API (Google Inc and Google Maps 2005) because we would like our system to be used anywhere. By using the Google Maps API, we can effectively acquire the parameters used in our system and visualize locations. Moreover, we can minimize the setup time required to obtain the cost parameters, such as distance and transit time, between nodes. We specifically use GDirections of the Google Maps API version 2, which requires approximately 1 s to acquire the parameters for each combination of nodes (Mizuno et al. 2012). We use either the transit time or distance as the distance parameter for the gravity model. The transit time and distance are used to confirm the tendency of the direction for users in the gravity model.

Using the method listed in Fig. 3, we obtain the parameters, distance, and transit time, which we store in the database, as illustrated in Table 4. In this table, Each From ID and To ID is a unique id for all combinations for each node. Once these parameters are saved in the database, we use them for the computations repeatedly.

**Table 4:** Example for information data for the nodes used in our system

ID	From ID	To ID	Distance(m)	Time (s)
1	1	2	6781	747
2	1	3	7314	868
3	1	4	8289	1314
4	1	5	16,396	1366
5	1	6	9175	1228
6	1	7	8002	1476
7	1	8	14,962	1041

### Optimization parameters

Next, we set the optimization parameters. In our approach, we perform optimization using the genetic algorithm (GA) (Cantù-Paz 1998; Zhang et al. 1999). As illustrated in Tables 5 and 6, the optimization parameters used are the number of genes, number of generations, number of bikes, total number of nodes available, service rate, and capacity of the number of bikes.

**Table 5:** Settings parameters for GA

Gene item	Value
Number of genes	100
Number of generations	1000
Intersection	Partially matched crossover
Selection pressure	0.7
Sudden generation	Insertion mutation
Sudden incidence	0.03
Parallelization method	Master–slave parallelization

**Table 6:** Parameters for the closed queueing network

Parameter	Value
Number of bikes	100
Total number of nodes	20
Service rate	5.0
Capacity of the number of bikes at each node	10

### Optimization procedure

**Fig. 4:**
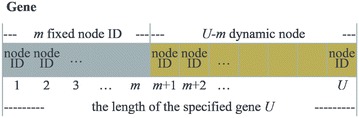
Composition of a gene

First, we select the number of nodes in the bike distribution system. There are two types of nodes: fixed and dynamic. We set *U* to be the total number of nodes that are candidates for optimization ($U\,\le\,K$). If we select *m* fixed nodes, the number of dynamic nodes is $U - m$. Then we create the gene with length *U* and each element has a node ID. Additionally, the system consists of *m* fixed nodes and $U - m$ dynamic nodes, as shown in Fig. 4.

We may need to analyze a varying number of fixed nodes. After we select the fixed nodes, we select the best nodes from the dynamic nodes to compute the objective function using a closed queueing network. Thus, we obtain the average queue length at each node. We should confirm the value of the objective function because we want to verify that the GA converges to plot the change of value for the objective function. Next, we display the results on Google Maps.

## Numerical example

We use regional information from Hamamatsu, Japan. In this region, there are 466 postal codes. In addition, we register as nodes the 304 convenience stores in Hamamatsu. In this case, we ignore the elevation of each node, to simplify the computation. We take the population-located nodes *i* and *j* as $q_{i,j}$ and $r_{j,i}$.

The settings of the GA used in this example are shown in Table 5. Several parallel computing techniques for GAs have been proposed (Darrell 1994). In this example, we adopt the master–slave parallelization approach because it is easily implemented in Google Maps using the PHP language. In Table 6, we show the parameters for the gravity model and closed queueing network.

The condition of capacity event $A_k$ for each node in (4) is as follows: the difference between the number of bikes and the capacity of a node is not larger than twice the node capacity, and is not less than 1/10 of its capacity. How to satisfy a condition such as this can be selected based the particular target model.

The GA minimizes the following objective function:

- For $$k = 1, 2, \ldots ,K$$,
- if $L_k > 2 \cdot CP_k$, then add $PT_K = 1000$ to the objective function,
- else if $L_k < CP_k \cdot 0.1$, then add $PT_k = 200$ to the objective function,
- else add $\left| L_k - CP_k \right|$ to the objective function.

### Decision for the gravity parameter

**Fig. 5:**
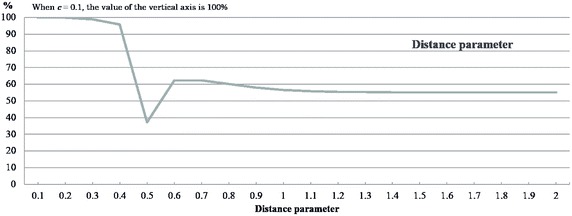
Value of the objective function while varying the distance parameter

We use the population parameters $\alpha$ and $\beta$, $\gamma$ and the distance parameter $\eta$ from (3). These can be determined flexibly according to the model being applied or the scenario under consideration. As an example of how to set these parameters, we proceed as follows: using the population in an area, the distance or transit time between nodes, and population transit time parameter, we employ the gravity model to obtain the transition probabilities (Ooyama 1993). Using the gravity model, we obtain parameters $\alpha$, $\beta$, $\gamma$ and $\eta$ from (3). In Fig. 5, we show the plot of the values of the objective function using 10 nodes, while varying the distance parameter. In this plot, the horizontal axis represents the value of the distance parameter. We observe that the value of the objective function changes as we vary the distance parameter. In this case, we concluded that the distance parameter should be set to 0.5 because this value minimized the objective function, as shown in Table 7.

**Table 7:** Parameters for the gravity model

Parameter	Value
Population parameter of the gravity model $\alpha$	1.0
Population parameter of the gravity model $\beta$	1.0
Population parameter of the gravity model $\gamma$	0.0
Distance parameter of the gravity model $\eta$	0.5

### Computing the initial number of fixed nodes

**Fig. 6:**
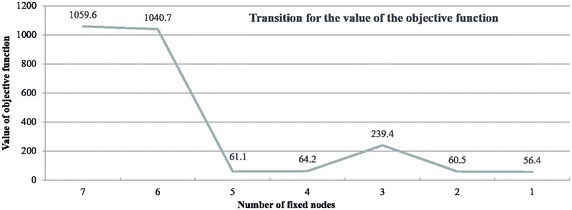
Value of the objective function while varying the number of fixed nodes

The number of fixed nodes plays an important role in node optimization. We examine the value of the objective function as we vary the number of fixed nodes. In Fig. 6, the horizontal axis represents the number of fixed nodes. Figure 6 demonstrates that we can obtain similar results using fewer fixed nodes. In the next sections, we present optimization results obtained for 20 nodes, of which only one is a fixed node.

### Optimization results for 20 nodes

**Fig. 7:**
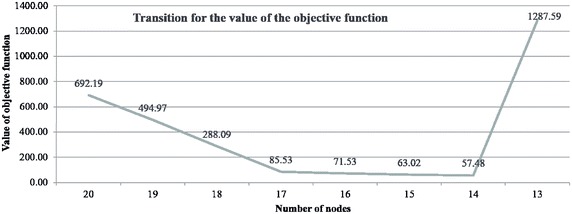
Value of the objective function when removing the node with the least number of bikes

We have the experiment of the previous section using 20 nodes. We use one fixed node and 19 dynamic nodes. The value of the objective function is 692.1918. Table 8, we observe three nodes, with node IDs 179, 232, and 247, that use <10% of the capacity of the number of bikes at each node. These nodes among the dynamic nodes are referred to as penalty nodes. We continue removing penalty nodes until there are no penalty nodes remaining. The results obtained are presented in Table 9. In this case, the value of the objective function is 85.5264, as shown in Fig. 7.

**Table 8:** Optimization results for 20 nodes

Node ID	Number of bikes	Element of the objective function for each node
15	5.722361	4.277639
23	2.039668	7.960332
25	6.334638	3.665362
34	2.590727	7.409273
42	12.10129	2.101291
48	8.77132	1.22868
56	6.257035	3.742965
57	1.888292	8.111708
65	3.077918	6.922082
79	4.900534	5.099466
81	7.030343	2.969657
112	1.693075	8.306925
171	1.141893	8.858107
175	18.00817	8.008174
179	0.752507	200
209	7.62364	2.37636
213	7.060485	2.939515
232	0.425842	200
247	0.794523	200
294	1.785734	8.214266

**Table 9:** The 17 optimized nodes obtained after removing the penalty nodes

Node ID	Number of bikes	Element of the objective function for each node
15	6.61131	3.38869
23	2.252811	7.747189
25	6.852832	3.147168
34	2.709976	7.290024
42	16.18106	6.181063
48	10.05988	0.05988
56	7.637594	2.362406
57	1.978323	8.021677
65	3.124947	6.875053
79	5.043066	4.956934
81	8.121089	1.878911
112	1.529695	8.470305
171	1.140414	8.859586
175	11.52231	1.522305
209	7.805536	2.194464
213	5.894068	4.105932
294	7.981989	2.018011

**Fig. 8:**
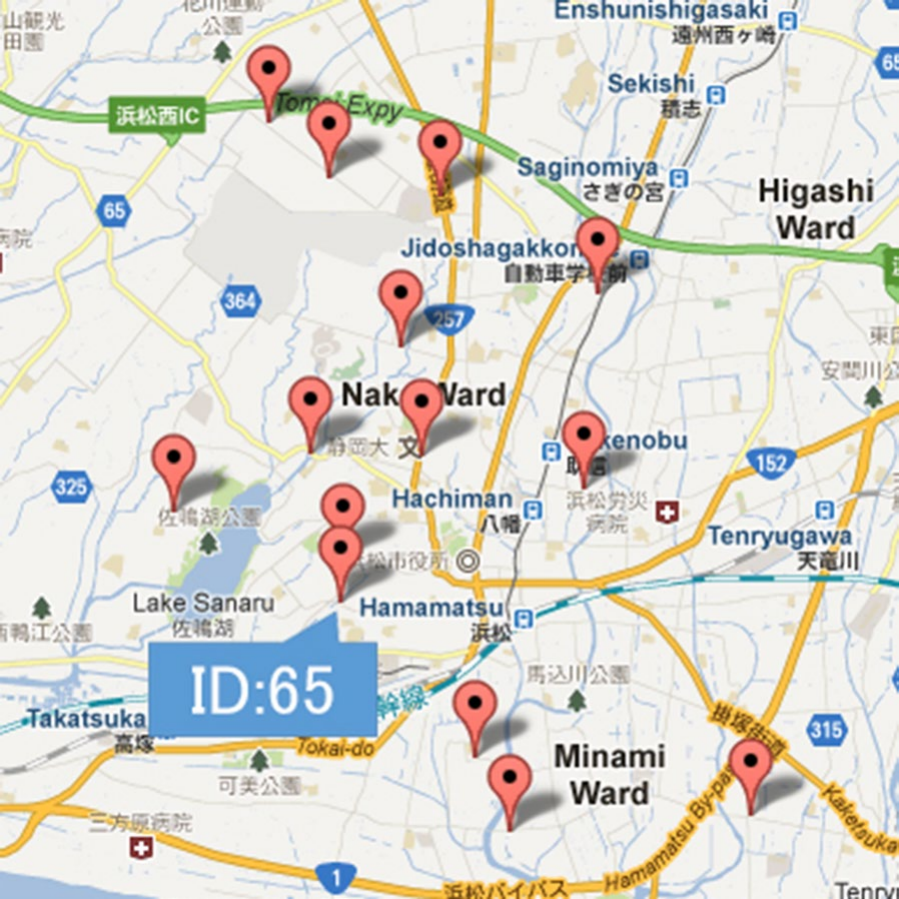
Locations of the 14 optimized nodes obtained by removing the nodes with the least number of bikes

Moreover, we continue removing the nodes for node IDs 112, 171, and 294, which have the least number of bikes, and optimize again. From Table 10, Figs. 7 and 8, we observe that the best results occur when 14 nodes are used.

**Table 10:** Optimization results for 14 nodes

Node ID	Number of bikes	Element of the objective function for each node
15	6.053414	3.946586
48	7.028729	2.971271
23	2.57486	7.42514
57	2.271532	7.728468
25	8.127648	1.872352
34	3.183185	6.816815
42	15.97184	5.971837
56	9.570738	0.429262
79	4.771828	5.228172
81	11.44458	1.444584
65	3.630369	6.369631
175	11.32166	1.321662
209	9.896153	0.103847
213	4.153461	5.846539

For an actual rental cycle system, administrators often transport bikes to other nodes by truck because of converging bikes at a specific node. In this model, if the optimal node is not chosen, bikes will converge at one node. We use the objective function effectively and we succeed in distributing bikes. Thus, we obtain better results by adjusting the optimization procedure. We suggest that this approach should be followed when designing a bike distribution system.

## Conclusion

Currently, in various places, social experiments are being conducted concerning the sharing of regular or battery-assisted bicycles. To increase the effectiveness of these bike distribution systems, it is important to carefully arrange the distribution nodes of the system. In this study, we optimized the arrangement of nodes of Hamamatsu, Japan. If provided with regional information, such as postal codes, region names, and node information, our approach can be applied to other locations. Moreover, using the Google Maps API, we can compute the required parameters in a timely fashion.

In this rental bike system, we have the problem that bikes converge at a specific node. To resolve this problem, we prepared the node candidate information exceeding 300 and 422 regional information. From our numerical computation, we found that we obtained better results when using as few fixed nodes as possible. If we use a greater number of nodes, such as 20 nodes, we obtain a better result than GA when removing a node according to specific conditions. Then we consider that such a calculation condition is required to perform the optimal placement and arrangement of rental bikes.

Our proposed approach has several unique features. First, all parameter computations can be performed using cloud computing and the Google Maps API. Next, using the gravity model, we can compute the transition probabilities through population and distance. It is important to determine the transition probability of the queueing network, thus we conclude that it is effective to use the gravity model. Finally, we performed effective analysis using a queuing network. Based on the results, we are confident that our proposed approach can be used to generate effective arrangements of bike distribution nodes.

We have several directions for future work. In our optimization problem, we assumed that the capacity and service rate of each node have the same values. As such, we investigated realistic node data, for example, elevation, to determine appropriate parameters for computing the transition probability, and we expect the optimization to be more representative of actual results. We need to interview field staff and gather real data to improve the model. The model also does not include the travel time between nodes. In this study, we used a GA for optimization. A more precise calculation is likely needed to compare computing time with utility. We also consider a simulation of this model to be needed because of the amount of information, such as a comparison of the results of this model and simulation including travel time. We aim next to develop a more realistic model coupled with simulation data.
